# Associations Between Restrained, Emotional, and External Eating Behaviors and Obesity Among Saudi Adults: A Cross-Sectional Study

**DOI:** 10.3390/nu18040631

**Published:** 2026-02-14

**Authors:** Merfat Abdulrahman Almaghrabi, Areej Bawajeeh, Israa M. Shatwan, Manal Malibary, Shahad Alzhrani, Nouf Alamoudi, Jena Almadani, Salwa Albar

**Affiliations:** 1 Food and Nutrition Department, King Abdulaziz University, P.O. Box 80200, Jeddah 21589, Saudi Arabia; eshatwan@kau.edu.sa (I.M.S.); salzahrani1539@stu.kau.edu.sa (S.A.); nalamoudi0074@stu.kau.edu.sa (N.A.); jalmadani0001@stu.kau.edu.sa (J.A.); 2 Food, Nutrition and Lifestyle Unit, King Fahd Medical Research Center, King Abdulaziz University, Jeddah 3270, Saudi Arabia

**Keywords:** emotional eating, external eating, restrained eating, obesity, Saudi Arabia

## Abstract

Background and Aim: Obesity has reached alarming levels globally and across the Middle East. In Saudi Arabia, approximately one-third of Saudi adults have obesity, representing a major public health concern. Understanding behavioral factors underlying obesity is essential; therefore, this study aimed to investigate the association between eating styles and obesity indicators among Saudi adults and to examine gender differences in these associations. Methods: This cross-sectional study included 997 adult (405 males and 592 females) aged ≥ 18 years residing in Saudi Arabia. Data were collected using a self-administered questionnaire covering sociodemographic characteristics, anthropometric measures, health status. Eating behaviors were assessed using the validated Dutch Eating Behavior Questionnaire (DEBQ), which measures restrained, emotional, and external eating styles. Regression models were used to examine gender differences in mean eating style scores and to assess associations between eating styles, categorical BMI, and body fatness. Results: The mean age of participants was 37.05 ± 13.39 years. Significant gender differences were observed in BMI, body fatness, and physical activity levels. Females demonstrated higher restrained eating scores compared with males (β = −0.14; 95% CI: −0.24, −0.04; *p* = 0.008). Participants with obesity exhibited higher emotional eating scores, while external eating was more prevalent among underweight participants. Body fatness was significantly associated with emotional and external eating but not restrained eating. Conclusions: BMI status appears to be associated with specific eating styles, particularly unhealthy eating behaviors. These findings highlight the importance of behavioral-focused nutritional interventions that support eating regulation rather than emphasizing weight loss alone.

## 1. Introduction

Obesity has reached alarming levels globally and across the Middle East, with prevalence increasing significantly worldwide. In 2016, approximately 1.9 billion adults were classified as overweight, while 650 million were classified as obese, representing 39% and 19% of the global adult population, respectively [1]. According to the latest estimates from the World Health Organization, approximately one in eight adults worldwide live with obesity. More recent data indicate that around 2.5 billion adults are classified as overweight, and 890 million are obese, corresponding to 43% of the global adult population being overweight and about 16% living with obesity worldwide [2]. These figures reflect a clear upward trend in global obesity rates over the past decade.

In Saudi Arabia, obesity has emerged as a major public health concern. Recent national data indicate that approximately one-third of Saudi adults are classified as obese [3,4]. Notably, the prevalence of obesity is higher among women than men in Saudi Arabia [5,6]. Furthermore, gender-based disparities in obesity prevalence across the Middle East and North Africa region exceed 10 percentage points [7]. This gender gap is consistent with broader regional trends, where cultural and lifestyle factors place women at greater risk of excess weight gain [8]. Such statistics underscore the urgent public health challenge posed by obesity and highlight the need to investigate underlying behavioral factors contributing to this condition [9].

In addition to the interaction of genetic predisposition, sedentary lifestyle, and environmental factors, eating behaviors play a pivotal role in the development of obesity. Dysfunctional eating patterns are associated with a substantial proportion of mortality attributable to non-communicable diseases [10]. Overeating is widely recognized as a key behavioral factor contributing to obesity. The abundance and diversity of food available in the market have been identified as major drivers of overeating [11].

Emotional, external, and restrained eating are three maladaptive eating behaviors that have been identified as contributors to excessive food intake [12]. Emotional eating refers to the tendency to consume food in response to emotional states such as anger, fear, or anxiety [8]. External eating describes food consumption triggered by external cues, such as the sight or smell of food, independent of internal satiety signals [13]. Restrained eating involves the intentional restriction of food intake to maintain or control body weight and is often associated with long-term dieting [14]. Evidence suggests that individuals with stronger emotional or external eating behaviors are at increased risk of obesity and obesity-related cardiometabolic disorders, including diabetes, hypertension, and hyperlipidemia [15]. Conversely, a balanced approach to dieting may support weight management, whereas rigid dietary restraint is typically associated with episodes of overeating and adverse long-term effects on body weight [16]. However, recent studies indicate that dietary habits may be influenced by mental health status [17,18].

Although emotional, external, and restrained eating behaviors have traditionally been described as potential contributors to overeating and subsequent weight gain, recent research suggests that the relationship between eating behaviors and obesity may be bidirectional. Individuals with overweight or obesity may develop maladaptive eating patterns in response to psychological distress, body dissatisfaction, or impaired hunger and satiety regulation. Thus, eating behaviors may act both as contributors to and consequences of excess body weight. Understanding this complex interplay is essential for interpreting eating patterns within populations experiencing high obesity prevalence [19].

Although evidence on emotional eating in Saudi Arabia remains limited, recent studies have reported that more than half of young Saudi women experienced moderate to high levels of emotional eating during the COVID-19 pandemic [20]. Similarly, approximately 50–55% of female university students in the Qassim region were classified as moderate or high emotional eaters, with higher prevalence observed among those who were overweight or obese [21]. Notably, these studies focused exclusively on females, and there is a lack of comparable data on emotional eating among Saudi males, highlighting an important gap in the literature. Moreover, no national-level studies have examined the association between eating behaviors and obesity in Saudi Arabia. Accordingly, understanding eating-related behavioral patterns is essential, as these behaviors represent modifiable risk factors that can be targeted through obesity prevention and intervention strategies. Therefore, this study aims to investigate the association between eating behaviors as outcome variables and obesity indicators among Saudi adults and to explore gender differences in these associations.

## 2. Materials and Methods

### 2.1. Study Design and Participants

This cross-sectional study included adults aged 18 years and older who were recruited using a non-probability convenience sampling technique. The sample size was calculated using a two-proportion comparison formula, based on a global prevalence of emotional eating (p_1_) of 45% (0.45) [22] and a prevalence of obesity among Saudi adults (p_2_) of 38% (0.38) [23]. Assuming a significance level of α = 0.05 and a statistical power of 80%, the effect size was calculated as h = 2 × [arcsin(√0.45) − arcsin(√0.38)] ≈ 0.142. The corresponding Z-scores were Z_1−_α/_2_ = 1.96 and Z_1−_β = 0.84. Accordingly, the required sample size was calculated using the following formula: n = ((1.96 + 0.84)/0.142)^2^ × 2 ≈ 779 participants. After accounting for an anticipated attrition rate of 25%, a total sample size of 974 participants was required. Exclusion criteria included the presence of chronic diseases, pregnancy, and breastfeeding. Ethical approval for the study was obtained from the Ethics Committee of King Abdulaziz University, Unit of Biomedical Ethics (Ethical Reference No. 325-25).

### 2.2. Data Collation

Participants were invited to voluntarily complete an online, self-administered questionnaire using Google Forms. The questionnaire was distributed via email and social media platforms, including WhatsApp and Telegram groups. Electronic informed consent was obtained from all participants prior to survey participation, and the study was conducted in accordance with the principles of the Declaration of Helsinki.

### 2.3. Assessment of Sociodemographic, Anthropometric and Health Characteristics of Study Participants

Sociodemographic characteristics included age, gender, marital status (single, married, divorced, or widowed), educational level (diploma without high school, high school graduate or equivalent, bachelor’s degree, or postgraduate degree), employment status (student, employed, unemployed [self-employed], unemployed [seeking employment], unpaid/housewife, or retired), and monthly household income (<5000 SAR, 5000–10,999 SAR, 11,000–20,000 SAR, or >20,000 SAR).

Body mass index (BMI) was calculated using self-reported anthropometric data by dividing body weight in kilograms by height in meters squared (kg/m^2^) to determine obesity status. BMI was classified according to World Health Organization (WHO) adult guidelines as underweight (BMI < 18.5 kg/m^2^), normal weight (BMI 18.5–<25 kg/m^2^), overweight (BMI 25–<30 kg/m^2^), or obese (BMI ≥ 30 kg/m^2^) [24].

Body fatness percentage (BF%), is a continuous variable, was measured using the equation verified by Deurenberg: BF% = 1.20 × BMI + 0.23 × age − 10.8 × sex − 5.4 [25]. As part of the survey, self-perceived health status was assessed using a single self-reported item asking participants whether they considered their health to be good (yes/no). Diagnosed health conditions were assessed using a checklist of common physician-diagnosed diseases, with participants allowed to select more than one condition. Responses were analyzed as categorical variables, and no composite health score was constructed. In addition, a question on the frequency of physical activity was used to assess physical activity levels. With response options ranging from never to everyday, participants indicated how frequently they participated in physical exercise. Frequency of physical activity was analyzed as a categorical variable.

### 2.4. Eating Styles Assessment

The eating styles instrument was adapted from the previously published Dutch Eating Behaviour Questionnaire (DEBQ), developed by Van Strien, et al. [26], which is a well-established and validated tool for assessing eating behaviors. The questionnaire was translated into Arabic using forward–back translation procedures. The translated version was reviewed by a panel of experts, consisting of three nutritionists and one physician, who conducted content validation and cultural adaptation by evaluating its relevance, comprehensiveness, and clarity of the questionnaire. The adapted DEBQ was subsequently pilot-tested among 30 participants to assess the clarity and appropriateness of the wording. Data from the pilot study were not included in the final analysis. The questionnaire was administered in Arabic and demonstrated good to excellent internal consistency. Cronbach’s alpha values were 0.92 for emotional eating (13 items), 0.85 for external eating (10 items), and 0.86 for restrained eating (9 items).

The questionnaire comprised three subscales: restrained eating (10 items; e.g., “How often do you refuse food or drink offered because you are concerned about your weight?”), emotional eating (13 items; e.g., “Do you have a desire to eat when things are going against you or when things have gone wrong?”), and external eating (10 items; e.g., “If you see others eating, do you also have the desire to eat?”). Participants expressed their feelings and thoughts about each item on a 5-point Likert scale scored as follows: 1 = never, 2 = rarely, 3 = sometimes, 4 = often, and 5 = very often. The score for each eating style is the average of all the item scores for that particular scale, and as the DEBQ reflecting negative feelings, the higher score means higher level of restrained eating, emotional eating, external eating [27]. Responses were collected online and reviewed for completeness prior to analysis. The final dataset was cleaned by checking for missing values, outliers, and inconsistencies. All variables were coded prior to analysis, and the cleaned dataset was used for statistical analyses.

### 2.5. Statistical Analysis

Descriptive and summary statistics were used to describe the general characteristics of the study participants. Mean and standard deviations (±SD) were used for continuous variables, whereas frequency and percentage (N, %) were used to describe categorical variables. A Pearson Chi Square test was used to investigate the differences between genders in the categorical general characteristics. A two-sample *t*-test (a two-tailed test) was used to test differences between genders in the continuous characteristics. In addition, summary statistics for the mean and ±SD of eating styles (emotional eating, external eating, and restraint eating) were calculated and presented for the total sample and by gender.

Three ordinary least squares linear regression models were used to investigate the relationship between eating style scores (emotional, ex-ternal, and restricted eating) and obesity indicators (BMI and body fatness). Each eating type outcome was assessed using a separate regression model. BMI was used as a categorical predictor in all models, with the normal-weight group serving as the reference category. Model-1 was used to assess differences in the mean eating style between genders, adjusting for confounders (age, BMI categories and physical activity). Model-2 was used to identify the association between eating styles and BMI categories, as well as body fatness, after adjusting for confounders (age, and gender). Model-3 was applied to investigate the association between eating styles and BMI as categorical data and body fatness, after stratifying the data by gender and adjusting only for age and physical activity as a confounder. Normality of residuals was checked using Q–Q plots and the Shapiro–Wilk test. A statistical significance level of *p* < 0.05 was used and all analyses were carried out using Stata statistical software (Stata/IC 16.1) release 12 (Stata Corporation).

## 3. Results

### 3.1. Paricipents’ General Characteristics

As shown in Table 1, a total of 997 participants were included in the analysis, comprising 592 females and 405 males. The mean age of the study population was 37.05 ± 13.39 years, with a significant mean age difference of 6.4 ± 0.74 years between males and females (*p* < 0.001). Significant gender differences were also observed in anthropometric measures. On average, males were taller by 13.78 ± 1.32 cm and weighed 18.51 ± 2.27 kg more than females (*p* < 0.001).

Regarding BMI categories, 32.5% of participants were classified as having normal weight, 32.1% as overweight, and 28.9% as obese. Males had a significantly higher mean BMI (28.26 ± 5.46 kg/m^2^) than females (*p* < 0.001). In contrast, body fatness was significantly higher among females than males, with a mean difference of 10.86 (95% CI: −11.43, −10.29; *p* < 0.0001).

More than half of the participants were married (63%) and held a bachelor’s degree (60%). A monthly household income of 10,999–20,000 SAR was reported by 41% of participants. The majority of participants (77%) reported good health status, with a significant difference between genders (*p* < 0.001). In terms of physical activity, 37.5% of participants reported rarely engaging in physical activity, followed by 22.6% who exercised once to twice per week; significant gender differences were observed (*p* < 0.001).

### 3.2. Participants’ Eating Styles by Total Sample and by Genders

Gender was identified as a significant predictor of restrained eating behaviour, with females reporting significantly higher restrained eating scores than males (β = −0.14, 95% CI: −0.24 to −0.04; *p* = 0.008). In contrast, the regression model showed no significant associations between gender and the other eating styles, namely emotional eating and external eating (Table 2).

### 3.3. Association Between Eating Styles with Obesity of Study Participants by Total Sample and Genders

As presented in Table 3, significant associations were observed between obesity indicators and eating styles. With respect to BMI categories, significant differences were found for emotional eating (*p* = 0.001) and external eating (*p* = 0.017). Participants classified as obese and underweight reported higher mean emotional eating scores (1.58 ± 0.73 and 1.52 ± 0.68, respectively) compared with those of normal weight. Similarly, underweight and obese participants demonstrated higher mean external eating scores (1.88 ± 0.81 and 1.76 ± 0.78, respectively) than normal-weight participants. Body fatness was significantly associated with emotional eating (β = 0.01, 95% CI: 0.01–0.02; *p* < 0.001) and external eating (β = 0.01, 95% CI: 0.01–0.02; *p* = 0.007), whereas no statistically significant association was observed with restrained eating.

Gender-stratified analyses revealed distinct patterns. Among females, BMI categories were significantly associated with emotional eating (*p* = 0.009), with obese females reporting higher mean emotional eating scores (1.59 ± 0.69). In addition, body fatness was significantly associated with emotional eating among females (β = 0.01, 95% CI: 0.01–0.02; *p* = 0.015). In contrast, among males, BMI categories were significantly associated with external eating (*p* = 0.019) and restrained eating (*p* = 0.026). Underweight males reported higher mean external eating scores (2.11 ± 1.01), while obese males exhibited higher mean restrained eating scores (1.73 ± 0.82). Furthermore, body fatness among males was significantly associated with emotional eating (β = 0.01, 95% CI: 0.01–0.02; *p* = 0.012), external eating (β = 0.02, 95% CI: 0.01–0.03; *p* = 0.005), and restrained eating (β = 0.02, 95% CI: 0.01–0.03; *p* = 0.003).

## 4. Discussion

There has been increasing interest in the literature in examining the association between obesity indicators and eating behaviors, such as emotional eating. Accordingly, this study investigated the relationship between eating styles and obesity indicators among Saudi adults. The findings indicated that restrained eating was positively associated with higher BMI among males whereas emotional eating was associated with higher BMI among participants, with a more pronounced effect observed among females. In addition, external eating was associated with underweight males. Body fatness was found to be significantly associated with both emotional eating and external eating.

Consistent with our findings, a previous study conducted among Lebanese adults reported that female participants and higher BMI were associated with higher restrained eating scores [28]. These gender differences may be explained by variations in eating behaviors, as women tend to exhibit greater dieting tendencies for weight control, higher food cravings, and a greater susceptibility to eating disorder–related behaviors [29,30]. In the present study, however, a positive association between restrained eating and higher BMI was observed only among males. Similarly, a cross-sectional study among Chinese students found that individuals who experienced greater body dissatisfaction or overestimated their body weight were more likely to engage in restrained eating behaviors [31]. A positive association between restrained eating and BMI has also been reported among Polish adolescents [32]. Individuals with excess body weight may exhibit higher levels of cognitive restraint in an effort to control their weight. Conversely, overweight and obesity may also result from restrained eating, as individuals attempting to limit food intake may experience a loss of control, leading to overeating when cognitive restraint is disrupted by factors such as emotional distress or depression [33,34,35].

The present study supports and extends previous evidence demonstrating an association between emotional eating and obesity [8,36,37,38]. In addition, a recent study reported that obese women with higher emotional eating scores had greater energy intake [39]. Emotional eating—defined as overeating in response to negative or positive emotional states—has been identified as a significant risk factor for the development of overweight and obesity [40,41]. Individuals with overweight or obesity may exhibit less effective coping strategies for managing emotional distress; consequently, food may become more appealing and serve as a source of emotional relief [42,43]. Emotional eaters may also experience a reduced ability to distinguish between internal hunger and satiety cues, partly because stress can impair the regulation and monitoring of these internal signals [44]. Supporting this mechanism, a 12-month intervention study found that participants who reduced emotional eating were 1.7 times more likely to achieve weight loss compared with those who maintained high levels of emotional eating [45].

In the current findings, external eating was more prevalent among underweight participants, particularly among males. These findings are consistent with those reported among female Indonesian university students [46], and Japanese female university students [47], but differ from findings reported in studies conducted in Algeria [8] and Turkey [48]. These discrepancies may be attributed to methodological differences, as the Algerian study did not include underweight participants, while the Turkish study focused exclusively on a younger population of university students, thereby limiting comparability with the current study. A possible explanation for the observed findings is that underweight individuals may be more responsive to external food cues, such as the smell or taste of food, rather than internal hunger cues when consuming food. This association appeared to be more pronounced among males, potentially because females are more influenced by emotional eating and are more likely to eat in response to mood-related factors [49].

In addition, significant associations were observed between body fatness and both emotional eating and external eating. Few studies have examined obesity indicators beyond BMI. Two studies conducted among women reported an association between restrained eating and body fatness [50]. However, these studies employed different methods for assessing body fatness and included smaller sample sizes compared with the present study.

Healthcare initiatives should incorporate comprehensive counseling programs that include dietary education, psychological approaches (e.g., mindfulness and self-compassion), and personalized support to effectively reduce the likelihood of developing maladaptive eating behaviors, such as emotional eating, restrained eating, and external eating. Accordingly, healthcare services are recommended to adopt a multidisciplinary team approach involving dietitians, psychologists, and physicians, to effectively identify and manage eating-related behaviors. Additionally, there is a need for randomized controlled trials and longitudinal studies that evaluate the long-term effectiveness of dietary behavior change interventions that combine diet and exercise with psychological therapies, such as Acceptance and Commitment Therapy (ACT). ACT is a behavioral and cognitive therapy that promotes psychological flexibility and the use of mindfulness-based techniques to regulate emotions, thereby supporting weight management and the control of maladaptive eating behaviors. Moreover, clinical studies investigating potential mechanisms underlying the intercorrelation between eating behaviors and psychosocial difficulties are highly recommended.

Several limitations of this study should be acknowledged. First, the cross-sectional design precludes the establishment of causal relationships. Future research using longitudinal or experimental designs is therefore recommended to better examine causality. Second, the use of a non-probability convenience sample recruited through voluntary online participation, which may restrict the representativeness and generalizability of the findings beyond the study population. In addition, the online-based survey format may have limited the recruitment of older participants. Third, although data were collected using a validated questionnaire, self-reported measures are subject to potential reporting errors, including social desirability bias (where participants may underreport or overreport behaviors) and recall bias. Although the use of self-reported weight and height to calculate BMI represents a limitation, it is considered an acceptable method for determining BMI in large-scale population studies [51]. Also, a key drawback of this study is that no additional sociodemographic and health factors were included in the regression models. This represents a predetermined analytical choice based on the study’s aim and model parsimony, rather than exclusion based on statistical significance; hence, residual confounding cannot be ruled out. Having said that, overall health status was measured using a simple binary (0–1), which could be a problematic since it reduces varied health statuses to a single threshold. Future research should avoid using a simple binary (0–1) categorization to determine health status effect. Despite these limitations, this study has several strengths. Potential confounding variables were appropriately adjusted for in the statistical analyses, enhancing the robustness of the findings. Furthermore, the study sample size was adequate and determined based on prior sample size calculations, supporting the reliability of the results.

## 5. Conclusions

The findings of the present study demonstrate an association between BMI status and eating styles, particularly unhealthy eating behaviors. Obesity was associated with emotional eating, whereas underweight participants exhibited higher levels of external eating. Gender-specific patterns were also observed, with females being more likely to overeat in response to emotional states, while males with obesity exhibited higher levels of restrained eating. These findings highlight the importance of providing individuals exhibiting unhealthy eating behaviors with targeted nutritional counseling or intervention programs that focus on regulating eating behaviors rather than solely emphasizing weight loss.

One promising approach is Acceptance and Commitment Therapy (ACT), an intervention model that integrates acceptance and mindfulness strategies with commitment and behavior-change techniques [52]. ACT focuses on the functional role of thoughts and emotions in individuals’ daily lives and their relationship with these experiences, with the aim of enhancing psychological flexibility and supporting healthier regulation of behavior.

## Figures and Tables

**Table 1:** General characteristics of the study participants by total sample and gender.

General Characteristics	All Participants (n = 997)	Females (n = 592)	Males (n = 405)	*p*-Value
Age (years) (mean ± SD) *	37.05 (±13.39)	34.46 (±12.71)	40.86 (±13.45)	<0.001
Height (cm) (mean ± SD) *	163.98 (±9.58)	158.38 (±6.21)	172.16 (±7.53)	<0.001
Weight (kg) (mean ± SD) *	72.96 (±19.19)	65.44 (±15.95)	83.95 (±18.22)	<0.001
BMI (kg/m^2^) (mean ± SD) *	26.97 (±5.98)	26.09 (±6.16)	28.26 (±5.46)	<0.001
Body fatness (mean ± SD) *	31.09 (±9.27)	33.83 (±9.18)	27.11 (±7.85)	<0.001
**BMI categories (kg/m^2^)** (N, %) ^†^				
Underweight	65 (6.52%)	52 (8.78%)	13 (3.21%)	<0.001
Normal weight	324 (32.50%)	231 (39.02%)	93 (22.96%)	
Overweight	320 (32.10%)	162 (27.36%)	158 (39.01%)	
Obese	288 (28.89%)	147 (24.83%)	141 (34.81%)	
**Social status** (N, %) ^†^				
Single	320 (32.10%)	224 (37.84%)	96 (23.70%)	<0.001
Married	625 (62.69%)	326 (55.07%)	299 (73.83%)	
Divorced	32 (3.21%)	26 (4.39%)	6 (1.48%)	
Widowed	20 (2.01%)	16 (2.70%)	4 (0.99%)	
**Education level** (N, %) ^†^				
Diploma	81 (8.12%)	43 (7.26%)	38 (9.38%)	0.057
High school	232 (23.27%)	134 (22.64%)	98 (24.20%)	
Bachelor’s degree	602 (60.38%)	375 (63.34%)	227 (56.05%)	
Postgraduate degree	82 (8.22%)	40 (6.76%)	42 (10.37%)	
**Economic status (for family)** (N, %) ^†^				
Less than 5000 riyals	147 (14.74%)	100 (16.89%)	47 (11.60%)	<0.001
5000–10,000 riyals	299 (29.99%)	211 (35.64%)	88 (21.73%)	
10,999–20,000 riyals	411 (41.22%)	215 (36.32%)	196 (48.40%)	
More than 20,999 riyals	140 (14.04%)	66 (11.15%)	74 (18.27%)	
**Health status (yes)** (N, %) ^†^	770 (77.23%)	420 (70.95%)	350 (86.42%)	<0.001
**Physical activity level** (N, %) ^†^				
Never	114 (11.43%)	75 (12.67%)	39 (9.63%)	<0.001
Rarely	374 (37.51%)	266 (44.93%)	108 (26.67%)	
1–2 times per week	225 (22.57%)	120 (20.27%)	105 (25.93%)	
3–4 times per week	168 (16.85%)	87 (14.70%)	81 (20.00%)	
daily	116 (11.63%)	44 (7.43%)	72 (17.78%)	

* Independent-samples *t*-tests were used to compare continuous variables between males and females, which are shown as mean ± SD; ^†^ Chi-square tests were used to compare categorical variables, which are displayed as counts (%).

**Table 2:** Eating styles (mean ± SD) and their associations with gender.

Eating Styles	All Participants (n = 997) Mean (±SD)	Females (n = 592) Mean (±SD)	Males (n = 405) Mean (±SD)	Coef. 95% CI	*p* Value ^†^
Emotional eating	1.47 (±0.62)	1.48 (±0.62)	1.46 (±0.61)	−0.01 (−0.09, 0.08)	0.897
External eating	1.69 (±0.72)	1.69 (±0.73)	1.70 (±0.71	0.06 (−0.04, 0.15)	0.243
Restraint eating	1.71 (±0.76)	1.74 (±0.75)	1.67 (±0.77)	−0.14 (−0.24, −0.04)	0.008

The values are presented as unadjusted means (SD) derived from raw data. Each eating style score (emotional, external, and restricted eating) was employed as a dependent variable in different ordinary least squares linear regression models, with gender serving as the independent variable. The regression coefficients (95% CI) and realted *p*-values represent the values that estimate the relationship between gender and each eating style score. Eating styles were measured using the DEBQ. ^†^ Model1: adjusted for age, BMI categorical data and physical activity.

**Table 3:** Associations between eating style scores and BMI catgories and body fatness in the total sample and stratified by gender.

Obesity Indicator	Emotional Eating		*p* Value	External Eating		*p* Value	Restraint Eating		*p* Value
	Mean ± SD	Coef. 95% CI		Mean ± SD	Coef. 95% CI		Mean ± SD	Coef. 95% CI	
Total sample ^†^									
BMI (kg/m^2^)									
Under weight	1.52 ± 0.68	0.07 (−0.1, 0.24)	<0.001	1.88 ± 0.81	0.14 (−0.05, 0.34)	0.017	1.56 ± 0.75	−0.12 (−0.33, 0.08)	0.153
Normal weight (ref)	1.41 ± 0.56	-		1.68 ± 0.71	-		1.68 ± 0.73	-	
Overweight	1.43 ± 0.54	0.06 (−0.04, 0.16)		1.61 ± 0.65	−0.01 (−0.12, 0.11)		1.77 ± 0.79	0.11 (−0.02, 0.23)	
Obese	1.58 ± 0.73	0.21 (0.11, 0.32)		1.76 ± 0.78	0.15 (0.03, 0.27)		1.71 ± 0.75	0.05 (−0.08, 0.18)	
Body fatness	31.09 ± 9.27	0.01 (0.01, 0.02)	<0.001	-	0.01 (0.01, 0.02)	0.007	-	0.01 (−0.01, 0.01)	0.152
Females ^‡^									
BMI (kg/m^2^)									
Under weight	1.55 ± 0.74	0.13 (−0.06, 0.32)	0.009	1.83 ± 0.76	0.09 (−0.13, 0.31)	0.434	1.63 ± 0.81	−0.08 (−0.31, 0.15)	0.476
Normal weight (ref)	1.39 ± 0.52	-		1.69 ± 0.73	-		1.74 ± 0.73	-	
Overweight	1.47 ± 0.63	0.11 (−0.02, 0.25)		1.61 ± 0.72	−0.03 (−0.19, 0.12)		1.82 ± 0.83	0.03 (−0.13, 0.19)	
Obese	1.59 ± 0.69	0.24 (0.10, 0.38)		1.72 ± 0.72	0.09 (−0.08, 0.25)		1.69 ± 0.69	−0.09 (−0.26, 0.08)	
Body fatness	33.83 ± 9.18	0.01 (0.01, 0.02)	0.015	-	0.01 (−0.01, 0.01)	0.273	-	−0.01 (−0.01, 0.01)	0.465
Males ^‡^									
BMI (kg/m^2^)									
Under weight	1.41 ± 0.39	0.11 (−047, 0.24)	0.051	2.11 ± 1.01	0.37 (−0.04, 0.78)	0.019	1.28 ± 0.33	−0.27 (−0.72, 0.18)	0.026
Normal weight (ref)	1.46 ± 0.63	-		1.63 ± 0.67	-		1.52 ± 0.72	-	
Overweight	1.38 ± 0.42	−0.04 (−0.19, 0.12)		1.61 ± 0.57	0.04 (−0.14, 0.23)		1.72 ± 0.75	0.22 (0.02, 0.42)	
Obese	1.56 ± 0.76	0.14 (−0.02, 0.31)		1.80 ± 0.81	0.23 (0.05, 0.42)		1.73 ± 0.82	0.23 (0.02, 0.43)	
Body fatness	27.11 ± 7.85	0.01 (0.01, 0.02)	0.012	-	0.02 (0.01, 0.03)	0.005	-	0.12 (0.01, 0.03)	0.003

The values are presented as unadjusted means (SD) derived from raw data. Ordinary least squares linear regression models were used to calculate regression coefficients (95% CI) and related *p*-values, with each eating style score (emotional, external, and restrained eating) as the dependent variable and BMI category or body fatness as the independent variable. Analyses were carried out on the entire sample (Model 2) and stratified by gender (Model 3). Eating styles were measured using the DEBQ. ^†^ Model 2 (total sample): associations between BMI category or body fatness and eating style scores were assessed using ordinary least squares linear regression, accounting for age and gender as covariates. ^‡^ Model 3 (gender-stratified): associations between BMI category or body fatness and eating style scores were assessed using ordinary least squares linear regression, stratified by gender, and adjusted for age and physical activity as covariates.

## Data Availability

The data presented in this study are available in the King Abdulaziz University or upon request from the corresponding author.
